# *AHP2*, *AHP3*, and *AHP5* act downstream of *CKI1* in Arabidopsis female gametophyte development

**DOI:** 10.1093/jxb/erx181

**Published:** 2017-06-13

**Authors:** Zhenning Liu, Li Yuan, Xiaoya Song, Xiaolin Yu, Venkatesan Sundaresan

**Affiliations:** 1 College of Agriculture and Forestry Sciences, Linyi University, Linyi, Shandong, China; 2 Department of Plant Biology, University of California, Davis, CA, USA; 3 Institute of Vegetable Sciences, Zhejiang University, Hangzhou, China

**Keywords:** Arabidopsis, female gametophyte development, histidine phosphotransfer proteins

## Abstract

Histidine phosphotransfer proteins (HPs) are key elements of the two-component signaling system, which act as a shuttle to transfer phosphorylation signals from histidine kinases (HKs) to response regulators (RRs). *CYTOKININ INDEPENDENT 1* (*CKI1*), a key regulator of central cell specification in the Arabidopsis female gametophyte, activates the cytokinin signaling pathway through the Arabidopsis histidine phosphotransfer proteins (AHPs). There are five *HP* genes in Arabidopsis, *AHP1*–*AHP5*, but it remains unknown which *AHP* genes act downstream of *CKI1* in Arabidopsis female gametophyte development. Promoter activity analysis of *AHP1–AHP5* in embryo sacs revealed *AHP1*, *AHP2*, *AHP3*, and *AHP5* expression in the central cell. Phenotypic studies of various combinations of *ahp* mutants showed that triple mutations in *AHP2*, *AHP3*, and *AHP5* resulted in defective embryo sac development. Using cell-specific single and double markers in the female gametophyte, the *ahp2-2 ahp3 ahp5-2/+* triple mutant ovules showed loss of central cell and antipodal cell fates and gain of egg cell or synergid cell attributes, resembling the *cki1* mutant phenotypes. These data suggest that *AHP2*, *AHP3*, and *AHP5* are the major factors acting downstream of *CKI1* in the two-component cytokinin signaling pathway to promote Arabidopsis female gametophyte development.

## Teaser

Only AHP2, AHP3, and AHP5 are downstream targets of CKI1 in regulating Arabidopsis female gametophyte development.

## Introduction

Two-component signaling systems were originally identified in bacteria, the simplest form of which employs a receptor kinase and a response regulator (RR). In response to an environmental stimulus, the histidine kinase (HK) perceives the signal and autophosphorylates itself on a conserved histidine residue, and the phosphate is then transferred to a conserved aspartic acid residue within another group of signal transducers called the RRs (Mizuno, 1997; Stock *et al.*, 2000). Plants have multistep phosphorelays involving HKs, histidine-containing phosphotransfer proteins (HPs), and RRs. The HPs are responsible for phosphate transfer from the HKs to the RRs, which could contribute to increasing diversity and complexity of the signal transduction. Multistep phosphorelays have been implicated in the growth and development regulation, hormone responses, and osmotic stresses of plants (Schaller, 2000; Maxwell and Kieber, 2010; Müller, 2011).

The five Arabidopsis *HP* genes, *AHP1–AHP5*, encode small proteins with putative histidine phosphotransfer activity similar to that of the yeast and prokaryotic histidine phosphotransfer domains. AHPs are localized in the cytoplasm and the nucleus, to transfer phosphorylation signals from AHKs to the ARRs (Arabidopsis RRs) (Hwang *et al.*, 2002). A sixth protein, AHP6, which lacks the conserved histidine residue, is an inhibitory pseudo-phosphotransfer protein that inhibits the phosphorelay from AHPs to ARRs (Mähönen *et al.*, 2006).

*CYTOKININ INDEPENDENT 1* (*CKI1*) was identified as a HK gene, which could induce a typical cytokinin response in the absence of cytokinin when overexpressed (Kakimoto, 1996). *CKI1* is primarily expressed in the micropylar end of embryo sacs, and loss-of-function mutants are semi-sterile and exhibit a block in megagametogenesis, mainly characterized by the abortion or degradation of embryo sacs (Pischke *et al.*, 2002; Hutchison *et al.*, 2006; Hejátko *et al.*, 2009). Research showed that the *CKI1*-induced cytokinin response is independent of cytokinin receptors but is dependent on *AHP1–AHP5* (Deng *et al.*, 2010), and further studies on *cki1/+* mutants and cytokinin receptor mutants indicated that the CKI1*–*AHPs*–*ARRs pathway, rather than the cytokinin receptor AHK*–*AHP*–*ARR pathway, is required for female gametophyte development (Pischke *et al.*, 2002; Hutchison *et al.*, 2006; Hejátko *et al.*, 2009; Deng *et al.*, 2010). Deng *et al.* (2010) showed that the *ahp1 ahp2 ahp3 ahp4 ahp5* quintuple mutants caused severe defects in female gametophyte development, the progression of female gametogenesis was disturbed, embryo sacs were arrested at the FG5 stage, or degraded to varying degrees, but the male gametophyte development was unaffected, which resembled the phenotypes described in *cki1* mutants (Pischke *et al.*, 2002; Hejátko *et al.*, 2003; Rabiger and Drews, 2013; Yuan *et al.*, 2016). However, the exact functions of AHPs involved in the CKI1*–*AHPs*–*ARRs pathway remain unknown. Protein*–*protein interactions between CKI1 and AHPs were assayed by bimolecular fluorescence complementation (BiFC), and the results showed that CKI1 interacts with AHP1, AHP2, AHP3, and AHP5; although a weak interaction signal was recorded for AHP1 (Pekárová *et al.*, 2011). However, in yeast two-hybrid assays, CKI1 interacted with only AHP2, AHP3, and AHP5, and not AHP1 (Urao *et al.*, 2000; Pekárová *et al.*, 2011). Thus, there is good evidence for AHP2, AHP3, and AHP5 interacting directly with CKI1, and the evidence for AHP1 as a CKI1 interactor remains ambiguous.

In this study, the promoter activities of *AHP1–AHP5* were primarily investigated in floral organs and embryo sacs. Various combinations of *ahp* mutants were constructed, and the morphological phenotypes were observed. Female gametophytic cell-specific markers were introduced to determine the cell fate switch in these mutants. Our results indicated that AHP2, AHP3, and AHP5 are downstream regulators of CKI1; these proteins act in an overlapping and redundant manner to regulate female gametophyte development, thereby providing more evidence to clarify the functions of the two-component system signaling network in plant reproduction.

## Materials and methods

### Plant materials and growth conditions

The *ahp1-1* (Ws, CS860143), *ahp2-2* (Col-0, SALK_019024), *ahp3* (Col-0, SALK_041384), *ahp5-2* (Col-0, SALK_079857), and *cki1-9*/+ (Col-0, SALK_057881) single mutants were obtained from the Arabidopsis Biological Resource Center (ABRC). The *ahp1-1 ahp2-2 ahp3/+ ahp4 ahp5-2* quintuple mutant was kindly provided by Jianru Zuo (Chinese Academy of Sciences). Plants were grown in a growth chamber with a 16 h light/8 h dark cycle at 22 **°**C with 60% relative humidity.

### Generation and screening of *ahp* multiple mutants

For the crosses, the flowers of the female parent were manually emasculated at 24 h before anthesis and cross-pollinated after 24 h. Double mutants were first generated by crossing single mutants with each other before double mutants were further crossed to generate triple mutants and quadruple mutants. Plants homozygous and heterozygous for insertions in all five loci were identified by PCR with gene-specific primers as described in Supplementary Table S1 at *JXB* online.

### Histology and microscopy

To observe seed development, siliques were dissected with needles and checked under a dissecting microscope. For whole-mount clearing observations, pistils containing at least 20 ovules were dissected and cleared overnight in Hoyer’s solution (Liu and Meinke, 1998). The ovules were subsequently observed under differential interference contrast (DIC) optics with a Zeiss 2 Axioplan imaging microscope (Axioskop 2 plus). For fluorescence microscopy, individual ovules were dissected from the pistils in 0.1 M phosphate buffer (pH 7.0), and samples were observed under a Zeiss 710 confocal laser scanning microscope (LSM 710). For β-glucuronidase (GUS) staining, inflorescences and four-whorl flower organs were dissected and incubated in the GUS staining buffer as previously described (Pagnussat *et al.*, 2007). Samples were observed under a Zeiss 2 Axioplan imaging microscope (Axioskop 2 plus).

### Constructs and plant transformation

For the *AHP1*, *AHP2*, *AHP3*, *AHP4*, *AHP5*, and *ETR1* promoter–reporter constructs, putative promoter regions (~2 kb upstream of ATG) were separately amplified from Col-0 genomic DNA. The PCR fragments were cloned into *pENTR/D-TOPO* (Invitrogen) and subsequently cloned into the gateway vector *pBGWFS7* and *NLS-3xGFP-NOST-pMLBART* by the LR clonase reaction (Invitrogen).

For the single cell-specific markers, the *Nos* terminator (NOST-*Hin*dIII-F/NOST-*Hin*dIII-R), *H2B* (H2B-*Xba*I-F/H2B-*Sal*I-R), and *eGFP* or *TagRFP* (*Sal*I-F/*Sal*I-R) were amplified by PCR and inserted into the *pCAMBIA1300* vector by a multiplestep process. The promoters *DD22*, *EC1.1*, *DD31*, and *DD13* (*Bam*HI-F/*Xba*I-R) were separately amplified from Col-0 genomic DNA and cloned into the *H2B-eGFP/TagRFP-NOST-pCMBIA1300* vector to generate the promoter–reporter constructs. To generate double cell-specific markers, fragments of promoter–*H2B*-*TagRFP*-*NOST* were amplified by PCR and cloned into single green fluorescent protein (GFP) marker constructs at the *Bam*HI cloning site.

Individual binary expression vectors were delivered into *Agrobacterium tumefaciens* AGL1, which was used to transform Col-0 wild-type plants or *ahp* multiple mutants. Transgenic plants were screened by spraying 1000-fold diluted glyphosate herbicide (Syngenta) or grown on Murashige and Skoog (MS) medium containing 25 mg l^–1^ hygromycin B (Invitrogen). At least 20 independent lines were assayed for each construct. For the marker transgenic plants, only lines showing Mendelian genetic segregation ratios were used.

## Results

### Expression of *AHP* genes in floral organs and embryo sacs

**Fig. 1.**
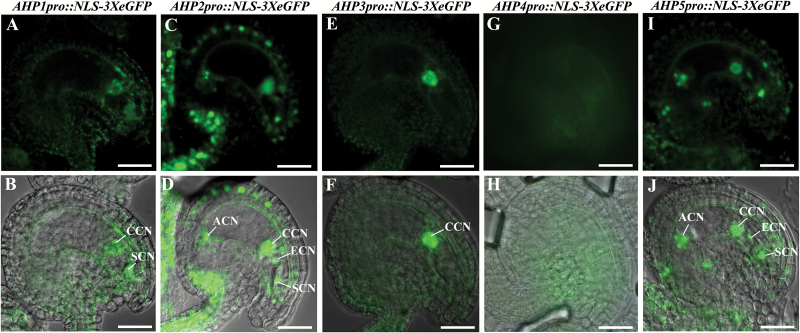
Expression of *AHP1*–*AHP5* in Arabidopsis mature embryo sacs. (A and B) *AHP1pro*::*NLS-3XeGFP*; (C and D) *AHP2pro*::*NLS-3XeGFP*; (E and F) *AHP3pro*::*NLS-3XeGFP*; (G and H) *AHP4pro*::*NLS-3XeGFP*; (I and J) *AHP5pro*::*NLS-3XeGFP*. CCN, central cell nucleus; SCN, synergid cell nuclei; ECN, egg cell nucleus; ACN, antipodal cell nuclei. Scale bars=20 µm.

*CKI1* expression has been reported in vascular tissues of floral organs and the chalazal domain of mature embryo sacs (Hejátko *et al.*, 2009; Yuan *et al.*, 2016). Genes acting downstream of *CKI1* might be expected to exhibit similar expression profiles. To investigate the expression patterns of *AHP1*–*AHP5*, we determined the transcriptional activity of transgenic lines with the *GUS* marker gene. GUS staining results showed that all five *AHP* genes exhibited vascular tissue-specific expression levels in the four whorls of floral organs (Supplementary Figs S1–S5). The expression profiles of these genes were further determined in the mature embryo sac by employing transgenic lines with the nuclear *eGFP* marker gene. The fluorescence signal of *AHP1* was detected in the central cell and the synergid cells; both *AHP2* and *AHP5* were expressed in all the female gametophytic cells; *AHP3* was specifically expressed in the central cell, whereas no signals were detected for *AHP4* in the embryo sac (Fig. 1).

### Silique check of *ahp* multiple mutants

Promoter activity analysis suggested that *AHP1*, *AHP2*, *AHP3*, and *AHP5* are candidate genes for regulation of female gametophyte development downstream of *CKI1*. As *ahp* single mutants are completely fertile, multiple mutant combinations were generated, and seed development was examined. As shown in Table 1 and Supplementary Fig. S6, the homozygous *ahp2-2 ahp3* double mutant, the *ahp2-2 ahp5-2* double mutant, and the *ahp3 ahp5-2* double mutant were fully fertile, similar to wild-type plants, without the obvious seed development abortion phenotype. Seed development in the homozygous *ahp1-1 ahp2-2 ahp3* triple mutant, the *ahp1-1 ahp2-2 ahp5* triple mutant, and the *ahp1-1 ahp3 ahp5-2* triple mutant was normal, also with no obvious differences from the control plant. However, the triple mutants with combinations of *ahp2-2*, *ahp3*, and *ahp5-2* showed obvious seed development abortion, characterized by unfertilized ovules. No homozygous triple mutants could be obtained, thereby suggesting the redundant functions among *AHP2*, *AHP3*, and *AHP5*. The ratio of unfertilized ovules in *ahp2-2 ahp3 ahp5-2/+* was 47.4% (*n*=430), whereas the ratios in *ahp2-2 ahp3/+ ahp5-2* and *ahp2-2/+ ahp3 ahp5-2* reached only 36.9% (*n*=452) and 36.3% (*n*=113), respectively. In addition, the *ahp1-1* allele was used for the construction of the quadruple mutants. The results showed that the ratio of unfertilized ovules in *ahp1-1 ahp2-2 ahp3 ahp5-2/+* was 47.2% (*n*=466), which was similar to that in *ahp2-2 ahp3 ahp5-2/+*. The ratios of unfertilized ovules in *ahp1-1 ahp2-2 ahp3/+ ahp5-2* and *ahp1-1 ahp2-2/+ ahp3 ahp5-2* were 35.5% (*n*=405) and 36.1% (*n*=432), respectively, which were similar to those in *ahp2-2 ahp3/+ ahp5-2* and *ahp2-2/+ ahp3 ahp5-2*. These results suggest that *AHP1* is not involved in the *CKI1* signaling pathway to regulate female gametophyte development. Similar observations in siliques with quadruple mutant combinations of *ahp4*, namely *ahp1-1 ahp2-2 ahp4 ahp5-2/+* and *ahp1-1 ahp2-2 ahp3/+ ahp4 ahp5-2*, also excluded the involvement of *AHP4* in this biological process.

**Table 1.** Silique check in *cki-9*/+ and *ahp* multiple mutants

Lines	Abnormal seeds	Normal seeds	Total
Wild type	6 (2.3%)	255 (97.7%)	261 (100%)
*cki1-9/+*	191 (48.8%)	200 (51.2%)	391 (100%)
*ahp2-2 ahp3*	5 (2.5%)	197 (97.5%)	202 (100%)
*ahp2-2 ahp5-2*	3 (1.3%)	230 (98.7%)	233 (100%)
*ahp3 ahp5-2*	7 (2.0%)	343 (98.0%)	350 (100%)
*ahp1 ahp2-2 ahp3*	10 (2.7%)	366 (97.3%)	376 (100%)
*ahp1 ahp2-2 ahp5*	5 (1.4%)	363 (98.6%)	368 (100%)
*ahp1 ahp3 ahp5-2*	8 (2.3%)	343 (97.7%)	351 (100%)
*ahp2-2 ahp3 ahp5-2/+*	204 (47.4%)	226 (52.6%)	430 (100%)
*ahp2-2 ahp3/+ ahp5-2*	167 (36.9%)	285 (63.1%)	452 (100%)
*ahp2-2/+ ahp3 ahp5-2*	41 (36.3%)	72 (63.7%)	113 (100%)
*ahp1 ahp2-2 ahp3 ahp5-2/+*	220 (47.2%)	246 (52.8%)	466 (100%)
*ahp1 ahp2-2 ahp3/+ ahp5-2*	144 (35.5%)	261 (64.4%)	405 (100%)
*ahp1 ahp2-2/+ ahp3 ahp5-2*	156 (36.1%)	276 (63.9%)	432 (100%)
*ahp1 ahp2-2 ahp4 ahp5-2/+*	7 (1.7%)	401 (98.3%)	408 (100%)
*ahp1 ahp2-2 ahp3/+ ahp4 ahp5-2*	98 (35.1%)	181 (64.9%)	279 (100%)

### Ovule clearing analysis of *ahp* multiple mutants

**Fig. 2.**
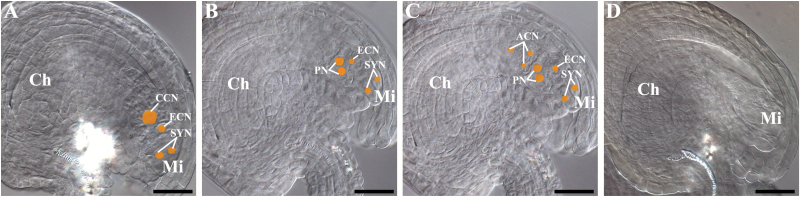
DIC microscopy of the cleared ovules in *ahp* multiple mutants. (A) Normal embryo sacs. (B–D) Abnormal embryo sacs. (B) Polar nuclei fail to fuse. (C) Polar nuclei fail to fuse; three antipodal cells persist; antipodal cell nuclei move toward the micropylar end. (D) Late stage degenerated embryo sacs with invisible female gametophyte nuclei. The position and size of the nuclei in embryo sacs are manually marked with yellow dots. Ch, chalazal end; Mi, micropylar end; CCN, central cell nucleus; ECN, egg cell nucleus; SYN, synergid cell nuclei; ACN, antipodal cell nuclei; PN, polar nuclei. Scale bars=15 µm.

To investigate further the underlying mechanism of the unfertilized ovules in *ahp* multiple mutants, ovules were cleared and observed. As shown in Fig. 2, Table 2, and Supplementary Fig. S7, compared with wild-type plants, 97.8% ovules showed normal embryo sacs without apparent phenotypic defects in the *ahp2-2 ahp3* double mutant plants. However, 46.2% of ovules (*n*=208) in *ahp2-2 ahp3 ahp5-2/+* showed abnormal embryo sacs; among which 32.2% of the polar nuclei were unable to fuse within the central cell, and 13.9% of the embryo sacs had degenerated by varying degrees. In *ahp2-2 ahp3/+ ahp5-2*, 31.6% of the ovules showed abnormal embryo sacs with unfused polar nuclei (18.7%) and degenerated embryo sacs (12.9%). Similarly, in *ahp2-2/+ ahp3 ahp5-2*, 30.6% of the ovules showed abnormal embryo sacs with unfused polar nuclei (18.5%) and degenerated embryo sacs (12.1%). Comparatively, these three mutants showed similar ratios of degenerated embryo sacs, but the ratio of unfused polar nuclei in *ahp2-2 ahp3 ahp5-2/+* was considerably higher than that in *ahp2-2 ahp3/+ ahp5-2* and *ahp2-2/+ ahp3 ahp5-2* (32.3% versus 18.7% and 18.5%, respectively). Notably, unlike the *cki1/+* mutants, the supernumerary nuclei phenotype was not observed in all three of these mutants. The ratios of abnormal embryo sacs by direct observations paralleled the unfertilized ovule ratios in corresponding *ahp* multiple mutants, indicating that unfertilized ovules might be attributable to the female gametophytic defects before fertilization.

**Table 2.** Classification of embryo sac phenotypes in *cki-9*/+ and *ahp* multiple mutants

Line	Abnormal embryo sacs				Normal embryo sacs	Total
	Unfused polar nuclei	Degarated embryo sacs	Supernumerary nuclei	Total		
Wild type	3 (2.1%)	2 (1.4%)	0 (0%)	5 (3.5%)	140 (96.5%)	145 (100%)
*cki1-9/+*	45 (33.8%)	16 (12.0%)	5 (3.8%)	66 (49.6%)	67 (50.4%)	133 (100%)
*ahp2-2 ahp3*	1 (0.4%)	4 (1.8%)	0 (0%)	5 (2.2%)	222 (97.8%)	227 (100%)
*ahp2-2 ahp3 ahp5-2/+*	67 (32.2%)	29 (13.9%)	0 (0%)	96 (46.2%)	112 (53.8%)	208 (100%)
*ahp2-2 ahp3/+ ahp5-2*	65 (18.7%)	45 (12.9%)	0 (0%)	110 (31.6%)	238 (68.4%)	348 (100%)
*ahp2-2/+ ahp3 ahp5-2*	55 (18.5%)	36 (12.1%)	0 (0%)	91 (30.6%)	206 (69.4)	297 (100%)

### Female gametophytic cell fates are altered in *ahp2-2 ahp3 ahp5-2/+* triple mutants

Since *ahp2-2 ahp3 ahp5-2/+*, *ahp2-2 ahp3/+ ahp5-2*, and *ahp2-2/+ ahp3 ahp5-2* mutants showed similar phenotypes under microscopy, the triple mutant *ahp2-2 ahp3 ahp5-2/+* was chosen as the representative mutant to study the female gametophytic cell fate specification.

**Fig. 3.**
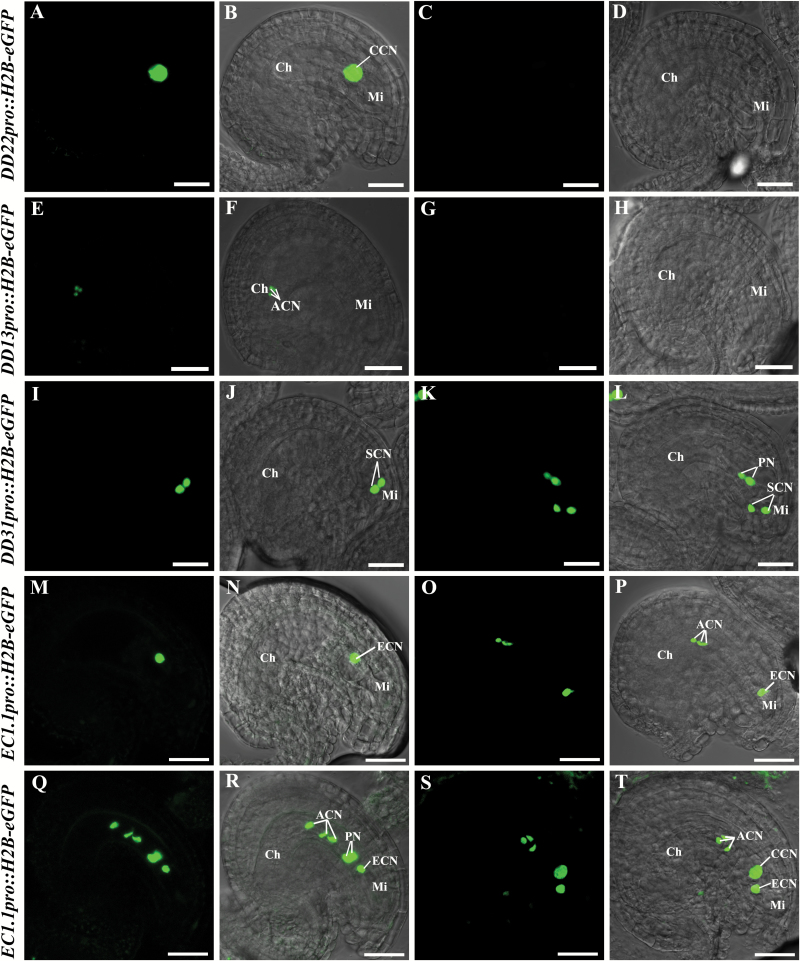
Expression of female gametophyte cell-specific markers in *ahp2-2 ahp3 ahp5-2/+* triple mutants. First and third column, nuclear-localized eGFP fluorescent signals; second column, merged images of the first column and the bright field images; fourth column, merged images of the third column and the bright field images. (A–D) Expression of a central cell-specific marker (*DD22pro::H2B-eGFP/+*). (A and B) Embryo sac with the central cell-specific marker; (C and D) embryo sac without the central cell-specific marker. (E–H) Expression of an antipodal cell-specific marker (*DD13pro::H2B-eGFP/+*). (E and F) Embryo sac with the antipodal cell-specific marker; (G and H) embryo sac without the antipodal cell-specific marker. (I–L) Expression of a synergid cell-specific marker (*DD31pro::H2B-eGFP*). (I and J) Embryo sac with the normal synergid cell-specific marker; (K and L) embryo sac with the abnormal synergid cell-specific marker; unfused polar nuclei also express the synergid cell-specific marker. (M–T) Expression of an egg cell-specific marker (*EC1.1pro::H2B-eGFP*). (M and N) Embryo sac with the normal egg cell-specific marker; (O–T) embryo sac with the abnormal egg cell-specific marker. (O and P) Three antipodal cells express the egg cell-specific marker. (Q and R) Three antipodal cells and two unfused polar nuclei all express the egg cell-specific marker. (S and T) Three antipodal cells and central cell express the egg cell-specific marker. Ch, chalazal end; Mi, micropylar end; CCN, central cell nucleus; ACN, antipodal cell nuclei; SCN, synergid cell nuclei; PN, polar nuclei; ECN, egg cell nuclei. Scale bars=20 µm.

Female gametophyte cell-specific single markers were primarily introduced into *ahp2-2 ahp3 ahp5-2/+* by the *Agrobacterium*-mediated floral dipping method. Thus, all plants examined were heterozygous for the markers, in addition to being heterozygous for *ahp5-2*. As shown in Fig. 3A–D and Table 3, the ratio of embryo sacs with normal central cell-specific positive GFP signals in *ahp2-2 ahp3 ahp5-2/+* was ~25% less than in the *ahp2-2 ahp3* control plant (25.2% versus 48.1%), thereby indicating the loss of the central cell fate in *ahp2-2 ahp3 ahp5-2/+*. Likewise, embryo sacs with normal antipodal cell-specific positive GFP signals in *ahp2-2 ahp3 ahp5-2/+* were also 25% less than in the *ahp2-2 ahp3* control plant (25.7% versus 50.0%) (Fig. 3E–H; Table 3), thereby indicating the loss of antipodal cell fate in *ahp2-2 ahp3 ahp5-2/+*. For synergid cell-specific marker expression, only 32.1% of the embryo sacs in *ahp2-2 ahp3 ahp5-2/+* showed normal GFP signals, whereas 16.5% of the embryo sacs showed that the two unfused polar nuclei also expressed the synergid cell-specific marker (Fig. 3I–L and Table 3), similar to the observed *cki1-9/+* phenotype (Yuan *et al.*, 2016), indicating the adoption of the synergid cell attributes in the polar nuclei. For egg cell-specific marker expression, additional phenotypic manifestations were observed. Only 26.0% of the embryo sacs in *ahp2-2 ahp3 ahp5-2/+* exhibited normal GFP signals; the remaining 21.9% of the embryo sacs exhibited the *cki1*-like phenotype of ectopic egg cell marker expression. Specifically, three antipodal cells expressed the egg cell-specific marker or three antipodal cells and two unfused polar nuclei expressed the egg cell-specific marker. Occasionally, the fused central cell also expressed the egg cell-specific marker (Fig. 3M–T; Table 3). These results demonstrated the adoption of the egg cell attributes in antipodal cells and unfused or fused polar nuclei.

**Table 3.** Expression of female gametophyte cell-specific markers in *ahp2 ahp3 ahp5/+* triple mutants

Lines	Positive GFP			Negative GFP	Total
	Positive GFP (wild type like)	Positive GFP (*cki1-9/+* like)	Total		
*ahp2 ahp3;* *DD22pro::H2B-eGFP/+*	90 (48.1%)	0 (0%)	90 (48.1%)	97 (51.9%)	187 (100%)
*ahp2 ahp3 ahp5/+;* *DD22pro::H2B-eGFP/+*	96 (25.2%)	0 (0%)	96 (25.2%)	285 (74.8%)	381 (100%)
*ahp2 ahp3;* *DD13pro::H2B-eGFP/+*	127 (50%)	0 (0%)	127 (50%)	127 (50%)	254 (100%)
*ahp2 ahp3 ahp5/+;* *DD13pro::H2B-eGFP/+*	83 (25.7%)	0 (0%)	83 (25.7%)	240 (74.3%)	323 (100%)
*ahp2 ahp3;* *DD31pro::H2B-eGFP/+*	138 (47.6%)	0 (0%)	138 (47.6%)	152 (52.4%)	290 (100%)
*ahp2 ahp3 ahp5/+;* *DD31pro::H2B-eGFP/+*	130 (32.1%)	67 (16.5%)	197 (48.6%)	208 (51.4%)	405 (100%)
*ahp2 ahp3;* *EC1.1pro::H2B-eGFP/+*	118 (47.2%)	0 (0%)	118 (47.2%)	132 (52.8%)	250 (100%)
*ahp2 ahp3 ahp5/+;* *EC1.1pro::H2B-eGFP/+*	88 (26.0%)	74 (21.9%)	162 (47.9%)	176 (52.1%)	338 (100%)

**Fig. 4.**
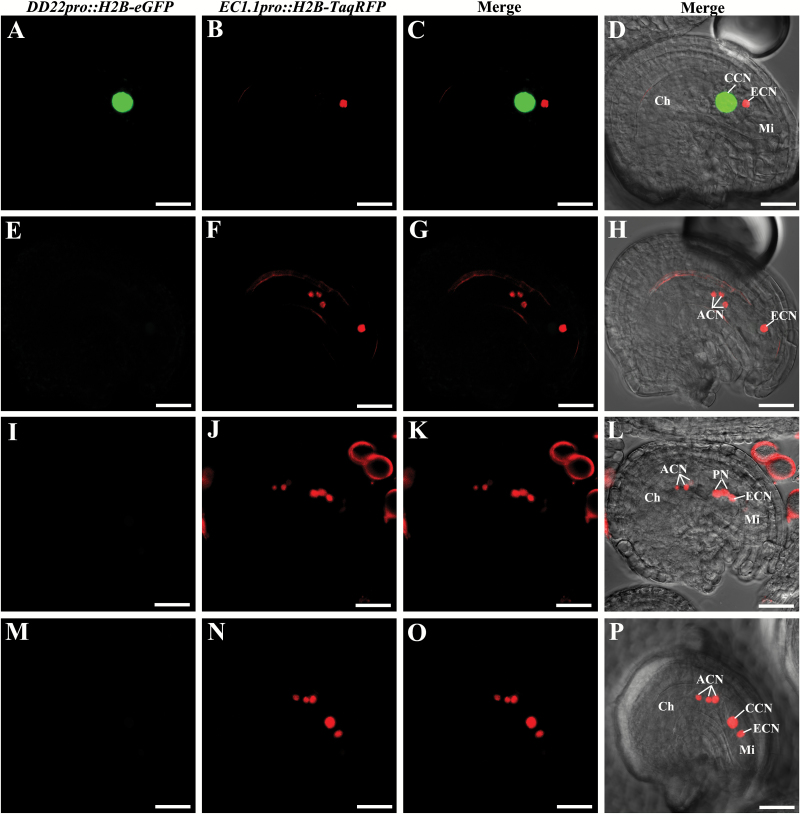
Expression of central cell-specific and egg cell-specific double markers (*DD22pro::H2B-eGFP* and *EC1.1pro::H2B-TagRFP*) in *ahp2 ahp3 ahp5/+* triple mutants. First column, central cell-specific marker expression; second column, egg cell-specific marker expression; third column, merged images of first and second columns; fourth column, merged images of the third column and the bright field images. (A–D) Embryo sacs with normal central cell-specific and egg cell-specific markers; (E–H) embryo sacs with abnormal central cell-specific and egg cell-specific markers; no central cell-specific markers were expressed in the central cell; egg cells show normal egg cell-specific markers. (E–H) Three antipodal cells showing egg cell-specific markers, (I–L) Three antipodal cells and two unfused polar nuclei showing egg cell-specific markers. (M–P) Three antipodal cells showing egg cell-specific markers, (I–L) Three antipodal cells and a central cell showing egg cell-specific markers. Ch, chalazal end; Mi, micropylar end; CCN, central cell nucleus; ECN, egg cell nucleus; PN, polar nuclei. Scale bars=20 µm.

**Fig. 5.**
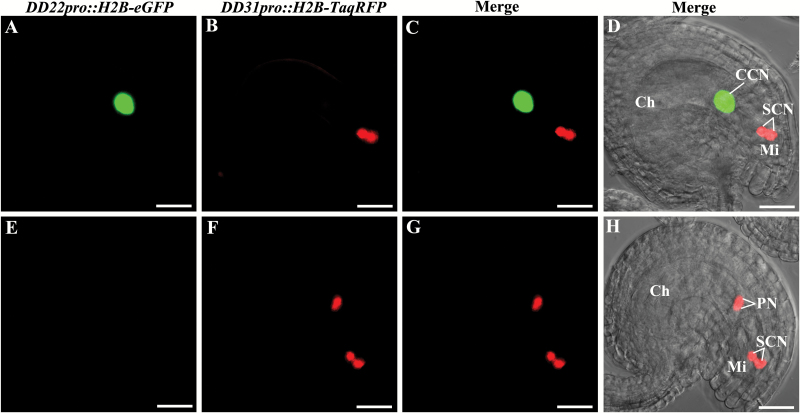
Expression of central cell-specific and synergid cell-specific double markers (*DD22pro::H2B-eGFP* and *DD31pro::H2B-TagRFP*) in *ahp2 ahp3 ahp5/+* triple mutants. First column, central cell-specific marker expression; second column, synergid cell-specific marker expression; third column, merged images of first and second columns; fourth column, merged images of the third column and the bright field images. (A–D) Embryo sacs with normal central cell-specific and synergid cell-specific markers. (E–H) Embryo sacs with abnormal central cell-specific and synergid cell-specific markers; two unfused polar nuclei do not show central cell-specific markers but show synergid cell-specific markers. Ch, chalazal end; Mi, micropylar end; CCN, central cell nucleus; SYN, synergid cell nuclei; PN, polar nuclei. Scale bars=20 µm.

Similar to *cki1* mutants (Yuan *et al.*, 2016), the central cell and antipodal cell fates might be expected to be transformed into the egg cell or synergid cell fates in *ahp2-2 ahp3 ahp5-2/+* triple mutants. Female gametophyte cell-specific double markers were used to confirm this hypothesis. For the egg cell-specific and central cell-specific double marker in the *ahp2-2 ahp3* control plants, 46.9% of the embryo sacs showed egg cell-specific red fluorescent protein (RFP) signals, and 47.7% of the embryo sacs showed central cell-specific GFP signals. These frequencies are as expected for normal embryo sac development. However, in the ovules of the *ahp2-2 ahp3 ahp5-2/+* triple mutant plants, only 26.2% of the embryo sacs showed normal egg cell-specific RFP signals and 24.1% of the embryo sacs showed normal central cell-specific GFP signals (Fig. 4A–D; Supplementary Table S2). Ectopic egg cell marker expression with concomitant loss of central cell marker expression was observed in 22.1% of the embryo sacs (Fig 4E–P). The ectopic egg cell expression with the double marker resembled the previously described expression observed with the egg cell-specific single marker (Fig. 3O–T). For the synergid cell-specific and central cell-specific double markers, two unfused polar nuclei showed synergid cell-specific RFP signals, whereas the central cell-specific GFP signal was negative (Fig. 5). These results provided more evidence that the central cell and antipodal cell fates were completely lost in *ahp2-2 ahp3 ahp5-2/+* embryo sacs, along with gain of egg cell or synergid cell fates. Thus, the *ahp2-2 ahp3 ahp5-2/+* triple mutants reproduced the phenotypes that were observed in *cki1* mutants, consistent with *AHP2*, *AHP3*, and *AHP5* acting downstream of *CKI1* to regulate cell fates in the chalazal domain of the embryo sac.

## Discussion

### Gene regulatory pathway for female gametophyte development

The supernumerary nuclei phenotype is characterized by the presence of 10–14 nuclei in embryo sacs, which exceeds the normal 8 nuclei. RETINOBLASTOMA RELATED (RBR) is known as a negative regulator of the cell cycle during mitosis; the loss of *RBR* perturbs the normal mitotic process, thereby leading to supernumerary nuclei because of the uncontrolled excessive nuclear division in embryo sacs (Ebel *et al.*, 2004; Johnston *et al.*, 2008). The supernumerary nuclei phenotype has also been identified in a small percentage of embryo sacs from the *cki1/+* mutant (Pischke *et al.*, 2002; Hejátko *et al.*, 2003; Rabiger and Drews, 2013; Yuan *et al.*, 2016), but its mechanism is still to be determined. In this study, we set out to determine which specific AHPs out of the five known AHPs in Arabidopsis act downstream of *CKI1* to regulate female gametophyte development. We determined that only *AHP2*, *AHP3*, and *AHP5* appear to be involved. Specifically, the *ahp2-2 ahp3 ahp5-2/+*, *ahp2-2 ahp3/+ ahp5-2*, and *ahp2-2/+ ahp3 ahp5-2* mutants all reproduced the *cki1/+* phenotype of defective cell type specification in the chalazal domain (i.e. specification of the central cell and antipodal cells). However, the supernumerary nuclei phenotype identified in a small percentage of embryo sacs in the *cki1/+* mutant (Pischke *et al.*, 2002; Hejátko *et al.*, 2003; Rabiger and Drews, 2013; Yuan *et al.*, 2016) was not observed with the *ahp2 ahp3 ahp5* triple mutants. This was also the case with various *ahp* quadruple combinations and the quintuple mutants (data not shown). It is possible that a CKI1-related regulatory mechanism distinct from the AHP–ARR pathway is responsible for the nuclear proliferation defect observed in a small fraction of *cki1* mutant embryo sacs.

### Involvement of AHP1 in the two-component signal transduction pathway

Promoter fusions of *AHP* genes showed that *AHP1*, *AHP2*, *AHP3*, and *AHP5* were expressed in embryo sacs, especially in the central cell. Functional analysis showed that only *AHP2*, *AHP3*, and *AHP5* contributed to normal embryo sac development, and an *ahp1* loss-of-function mutation did not enhance the *ahp2 ahp3 ahp5* triple mutant phenotype. Interestingly, in protein*–*protein interaction studies by yeast two-hybrid analysis, the ethylene receptor protein ETR1 was found to interact with three AHPs, AHP1*–*AHP3 (Urao *et al.*, 2000). Transcriptome expression data reveal *ETR1* expression in the central cell (Wuest *et al.*, 2010; Schmid *et al.*, 2012). The expression of *ETR1* in embryo sacs was further checked by promoter activity analysis. GUS staining and fluorescence microscopy results indicated that *ETR1* showed obvious expression in the central cell (Supplementary Fig. S8), which was consistent with transcriptome expression data (Wuest *et al.*, 2010; Schmid *et al.*, 2012). The ETR1*–*AHPs*–*ARRs ethylene signal transduction pathway seems independent of the classical ETR1*–*CTR1 ethylene signal transduction pathway (Chen *et al.*, 2005). Thus it is possible that *AHP1* might be involved in ethylene signaling through the ETR1*–*AHPs*–*ARRs signal transduction pathway; however, its specific functions in female gametophyte development still need to be clarified.

## Supplementary data

Supplementary data are available at *JXB* online.

Table S1. Summary of the primers used.

Table S2. Expresssion of the egg cell-specific and central cell-specific marker in *ahp2 ahp3 ahp5/+* triple mutants.

Fig. S1. *AHP1pro*::*GUS* expression in Arabidopsis floral organs.

Fig. S2. *AHP2pro*::*GUS* expression in Arabidopsis floral organs.

Fig. S3. *AHP3pro*::*GUS* expression in Arabidopsis floral organs.

Fig. S4. *AHP4pro*::*GUS* expression in Arabidopsis floral organs.

Fig. S5. *AHP5pro*::*GUS* expression in Arabidopsis floral organs.

Fig. S6. Microscopy of seed development in siliques.

Fig. S7. DIC microscopy of the cleared ovules in *ahp* multiple mutants.

Fig. S8. Transgenic expression in mature Arabidopsis embryo sacs.

## Author contributions

ZNL performed the experiments and analyzed the data, as well as drafting and writing the manuscript. YL and XYS contributed to mutant construction. VS and XLY proposed and supervised the research. All authors read and approved the final manuscript.

## Supplementary Material

Supplementary Figures S1-S8 and Tables S1-S2

## References

[CIT0001] Chen YF, Etheridge N, Schaller GE 2005 Ethylene signal transduction. Annals of Botany 95, 901–915. 15753119 10.1093/aob/mci100 PMC4246747

[CIT0002] Deng Y, Dong H, Mu J, Ren B, Zheng B, Ji Z, Yang WC, Liang Y, Zuo J 2010 *Arabidopsis* histidine kinase CKI1 acts upstream of histidine phosphotransfer proteins to regulate female gametophyte development and vegetative growth. The Plant Cell 22, 1232–1248. 20363773 10.1105/tpc.108.065128 PMC2879746

[CIT0003] Ebel C, Mariconti L, Gruissem W 2004 Plant retinoblastoma homologues control nuclear proliferation in the female gametophyte. Nature 429, 776–780. 15201912 10.1038/nature02637

[CIT0004] Hejátko J, Pernisová M, Eneva T, Palme K, Brzobohatý B 2003 The putative sensor histidine kinase CKI1 is involved in female gametophyte development in *Arabidopsis*. Molecular Genetics and Genomics 269, 443–453. 12774227 10.1007/s00438-003-0858-7

[CIT0005] Hejátko J, Ryu H, Kim GT 2009 The histidine kinases CYTOKININ-INDEPENDENT1 and ARABIDOPSIS HISTIDINE KINASE2 and 3 regulate vascular tissue development in *Arabidopsis* shoots. The Plant Cell 21, 2008–2021. 19622803 10.1105/tpc.109.066696 PMC2729606

[CIT0006] Hutchison CE, Li J, Argueso C 2006 The *Arabidopsis* histidine phosphotransfer proteins are redundant positive regulators of cytokinin signaling. The Plant Cell 18, 3073–3087. 17122069 10.1105/tpc.106.045674 PMC1693944

[CIT0007] Hwang I, Chen HC, Sheen J 2002 Two-component signal transduction pathways in *Arabidopsis*. Plant Physiology 129, 500–515. 12068096 10.1104/pp.005504 PMC161668

[CIT0008] Johnston AJ, Matveeva E, Kirioukhova O, Grossniklaus U, Gruissem W 2008 A dynamic reciprocal RBR–PRC2 regulatory circuit controls *Arabidopsis* gametophyte development. Current Biology 18, 1680–1686. 18976913 10.1016/j.cub.2008.09.026

[CIT0009] Kakimoto T 1996 CKI1, a histidine kinase homolog implicated in cytokinin signal transduction. Science 274, 982–985. 8875940 10.1126/science.274.5289.982

[CIT0010] Liu CM, Meinke DW 1998 The titan mutants of *Arabidopsis* are disrupted in mitosis and cell cycle control during seed development. The Plant Journal 16, 21–31. 9807824 10.1046/j.1365-313x.1998.00268.x

[CIT0011] Mähönen AP, Bishopp A, Higuchi M, Nieminen KM, Kinoshita K, Törmäkangas K, Ikeda Y, Oka A, Kakimoto T, Helariutta Y 2006 Cytokinin signaling and its inhibitor AHP6 regulate cell fate during vascular development. Science 311, 94–98. 16400151 10.1126/science.1118875

[CIT0012] Maxwell BB, Kieber JJ 2010 Cytokinin signal transduction. In: Davies PJ, ed. Plant hormones: biosynthesis, signal transduction, action! Dordrecht: Springer Netherlands, 329–357.

[CIT0013] Mizuno T 1997 Compilation of all genes encoding two-component phosphotransfer signal transducers in the genome of *Escherichia coli*. DNA Research 4, 161–168. 9205844 10.1093/dnares/4.2.161

[CIT0014] Müller B 2011 Generic signal-specific responses: cytokinin and context-dependent cellular responses. Journal of Experimental Botany 62, 3273–3288. 21212299 10.1093/jxb/erq420

[CIT0015] Pagnussat GC, Yu HJ, Sundaresan V 2007 Cell-fate switch of synergid to egg cell in *Arabidopsis**eostre* mutant embryo sacs arises from misexpression of the BEL1-like homeodomain gene *BLH1*. The Plant Cell 19, 3578–3592. 18055603 10.1105/tpc.107.054890 PMC2174879

[CIT0016] Pekárová B, Klumpler T, Třísková O 2011 Structure and binding specificity of the receiver domain of sensor histidine kinase CKI1 from *Arabidopsis thaliana*. The Plant Journal 67, 827–839. 21569135 10.1111/j.1365-313X.2011.04637.x

[CIT0017] Pischke MS, Jones LG, Otsuga D, Fernez DE, Drews GN, Sussman MR 2002 An *Arabidopsis* histidine kinase is essential for megagametogenesis. Proceedings of the National Academy of Sciences, USA 99, 15800–15805. 10.1073/pnas.232580499 PMC137796 12426401

[CIT0018] Rabiger DS, Drews GN 2013 MYB64 and MYB119 are required for cellularization and differentiation during female gametogenesis in *Arabidopsis thaliana*. PLoS Genetics 9, e1003783. 24068955 10.1371/journal.pgen.1003783 PMC3778002

[CIT0019] Schaller GE 2000 Histidine kinases and the role of two-component systems in plants. Advances in Botanical Research 32, 109–148.

[CIT0020] Schmid MW, Schmidt A, Klostermeier UC, Barann M, Rosenstiel P, Grossniklaus U 2012 A powerful method for transcriptional profiling of specific cell types in eukaryotes: laser-assisted microdissection and RNA sequencing. PLoS One 7, e29685. 22291893 10.1371/journal.pone.0029685 PMC3266888

[CIT0021] Stock AM, Robinson VL, Goudreau PN 2000 Two-component signal transduction. Annual Review of Biochemistry 69, 183–215. 10.1146/annurev.biochem.69.1.183 10966457

[CIT0022] Urao T, Miyata S, Yamaguchi-Shinozaki K, Shinozaki K 2000 Possible His to Asp phosphorelay signaling in an *Arabidopsis* two-component system. FEBS Letters 478, 227–232. 10930573 10.1016/s0014-5793(00)01860-3

[CIT0023] Wuest SE, Vijverberg K, Schmidt A, Weiss M, Gheyselinck J, Lohr M, Wellmer F, Rahnenführer J, von Mering C, Grossniklaus U 2010 *Arabidopsis* female gametophyte gene expression map reveals similarities between plant and animal gametes. Current Biology 20, 506–512. 20226671 10.1016/j.cub.2010.01.051

[CIT0024] Yuan L, Liu Z, Song X, Johnson C, Yu X, Sundaresan V 2016 The CKI1 histidine kinase specifies the female gametic precursor of the endosperm. Developmental Cell 37, 34–46. 27046830 10.1016/j.devcel.2016.03.009

