# Supplementary material for: AHP2, AHP3, and AHP5 act downstream of CKI1 in Arabidopsis female gametophyte development
Source: J Exp Bot. 2017 Jun 13;68(13):3365–73. doi: 10.1093/jxb/erx181 (PMC5853337; doi:10.1093/jxb/erx181)
Supplement: Supplementary Figures S1-S8 and Tables S1-S2 [file erx181_suppl_supplementary_figures_s1_s8_tables_s1_s2.pdf]

Supplementary tables and figures

Supplementary Table S1. Summary of the primers used.

Primer name	Primer sequences (5’-3’)	Purpose
BP-F	ATTTTGCCGATTTCGGAAC	Salk T-DNA insertion
JL202	CATTTTATAATAACGCTGCGGACATCTAC	*ahp1-1* T-DNA insertion
ahp1-1-LP	CAGAGAATATGGATTTGGTTCAGAAG	*ahp1-1* T-DNA insertion
ahp1-1-RP	ATGTGGGATTTAGATGTGGATTTAGAAAC	*ahp1-1* T-DNA insertion
ahp2-2-LP	AACATTATGGGCAAATTTGGG	*ahp2-2* T-DNA insertion
ahp2-2-RP	ATGTGAAGGGGTTTTTGGATG	*ahp2-2* T-DNA insertion
ahp3-LP	TCAAGCTGCAACAAAACAATG	*ahp3* T-DNA insertion
ahp3-RP	GGCTCTCCACAGTCTTTAGGG	*ahp3* T-DNA insertion
ahp5-2-LP	TTGGCTGGATTCTCACTTTTG	*ahp5-2* T-DNA insertion
ahp5-2-RP	TCAGAACAAAGGCTCCAAATG	*ahp5-2* T-DNA insertion
AHP1pro-Topo-F	CACCTTTGTCTATATGTCTACACACGAAG	*AHP1* promoter cloning
AHP1pro-Topo-R	ATTCTCTGTTATTTGCTTTTGTTCT	*AHP1* promoter cloning
AHP2pro-Topo-F	CACCTTCATTGCTGACTCTTTCGTCCTCC	*AHP2* promoter cloning
AHP2pro-Topo-R	GAGAAGAGTGAAGCGGAGATTGGGA	*AHP2* promoter cloning
AHP3pro-Topo-F	CACCTACTTTTCATCAATAGTTTGTTCTT	*AHP3* promoter cloning
AHP3pro-Topo-R	GGCTCTCTCTACTCGTCGTGAAGGC	*AHP3* promoter cloning
AHP4pro-Topo-F	CACCTAGAGGGGAGGTCGGAATTAAGGAG	*AHP4* promoter cloning
AHP4pro-Topo-R	GTTTGTTTAAATTTTGAGAATAGAT	*AHP4* promoter cloning
AHP5pro-Topo-F	CACCGAATCCCTTCTTTCATCCTCTCCAT	*AHP5* promoter cloning
AHP5pro-Topo-R	AGCTAAAGTTTACCAAGAACCAGAC	*AHP5* promoter cloning
NOST-*Hin*dIII-F	CCCAAGCTTGATCGTTCAAACATTTGGCAA	*NOST* cloning
NOST-*Hin*dIII-R	CCCAAGCTTATCGAATTCGATCTAGTAACATAGA	*NOST* cloning
H2B-*Xba*I-F	GCTCTAGAATGGCGAAGGCAGATAAGAA	*H2B* cloning
H2B-*Sal*I-R	GCGTCGACTCCTGATCCGGCTCCGGCACC	*H2B* cloning
eGFP-*Sal*I-F	GCGTCGACATGGTGAGCAAGGGCGAGGA	*eGFP* cloning
eGFP-*Sal*I-R	GCGTCGACTCATTGTTTGCCTCCCTGCTG	*eGFP* cloning

TaqRFP-*Sal*I-F	GCGTCGACATGGTGTCTAAGGGCGAAGAGCT	*TaqRFP* cloning
TaqRFP-*Sal*I-R	GCGTCGACTCAATTAAGTTTGTGCCCCAGTTTG	*TaqRFP* cloning
DD22pro-*Bam*HI-F	CGCGGATCCAGGTACCATATCCAGAAATGAGCT	*DD22* promoter cloning
DD22pro-*Xba*I-R	GCTCTAGAACTTTCACGTTTTCTTTTAAACTTT	*DD22* promoter cloning
EC1.1pro-*Bam*HI-F	CGCGGATCCAAACGCCTATCATGAATTAGCTCTA	*EC1.1* promoter cloning
EC1.1pro-*Xba*I-R	GCTCTAGATTCTCAACAGATTGATAAGGTCGAA	*EC1.1* promoter cloning
DD31pro-*Bam*HI-F	CGCGGATCCACCCACACGAAGAATCGGACT	*DD31* promoter cloning
DD31pro-*Xba*I-R	GCTCTAGATTTTTTTATGGATGTAAGAATACTT	*DD31* promoter cloning
DD13pro-*Bam*HI-F	CGCGGATCCTAAATGAGAGTACTATATTGTGGCA	*DD13* promoter cloning
DD13pro-*Xba*I-R	GCTCTAGATCTCAAAATCTGCATATATCTTTTT	*DD13* promoter cloning

Supplementary Table S2. Expresssion of egg cell-specific and central cell-specific marker in *ahp2 ahp3 ahp5/+* triple mutants.

**Lines**	**RFP signal**				**GFP signal**		**Total**
	**Embryo sacs with positive RFP**			**Embryo sacs with negative RFP**	**Embryo sacs with positive GFP**	**Embryo sacs with negative GFP**	
	**Positive RFP (WT like)**	**Positive RFP (*cki1-9/+* like)**	**Total**				
ahp2 ahp3; *EC1.1pro::H2B-eGFP&DD22pro::H2B-eGFP/+*	122 (46.9%)	0 (0%)	122 (46.9%)	138 (53.1%)	124 (47.7%)	136 (52.3%)	260 (100%)
ahp2 ahp3 ahp5/+; *EC1.1pro::H2B-eGFP&DD22pro::H2B-eGFP/+*	76 (26.2%)	64 (22.1%)	140 (48.3%)	150 (51.7%)	70 (24.1%)	220 (75.9%)	290 (100%)

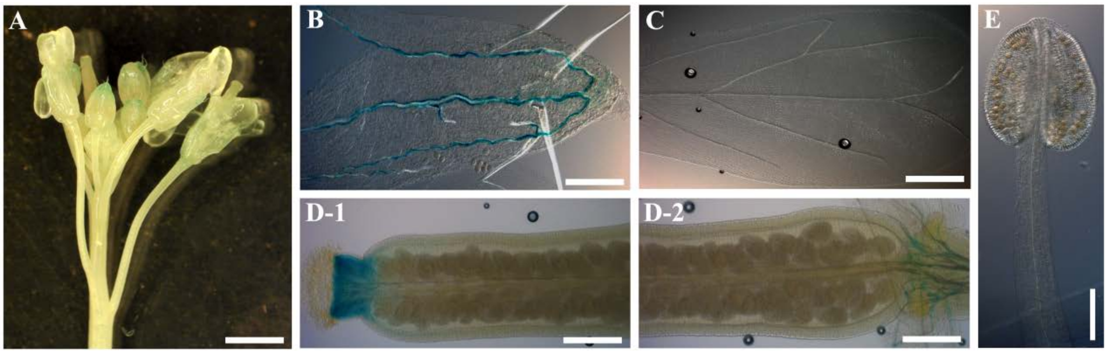

Supplementary Fig. S1. *AHP1pro*::*GUS* expression in Arabidopsis floral organs. (A) Inflorescence; (B) sepal; (C) petal; (D) pistil; (E) stamen. Scale bars: 2 mm (A), 200 µm (B–E).

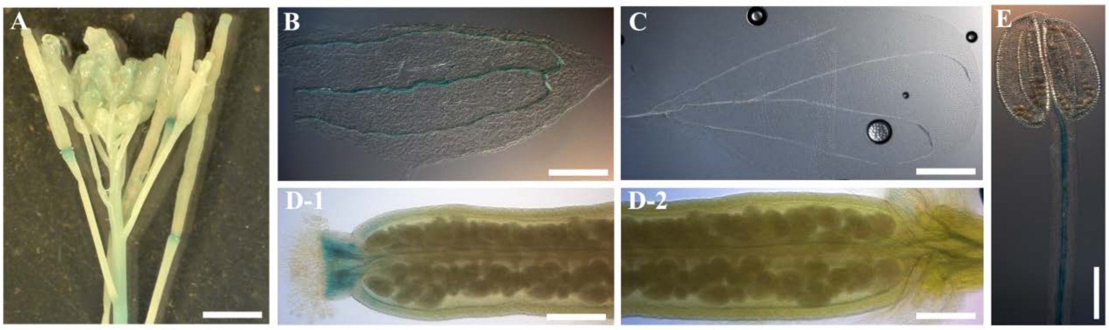

Supplementary Fig. S2. *AHP2pro*::*GUS* expression in Arabidopsis floral organs. (A) Inflorescence; (B) sepal; (C) petal; (D) pistil; (E) stamen. Scale bars: 2 mm (A), 200 µm (B–E).

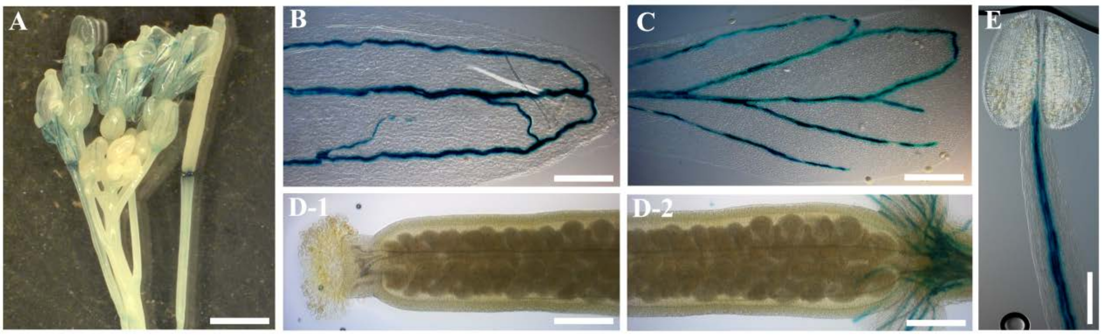

Supplementary Fig. S3. *AHP3pro*::*GUS* expression in Arabidopsis floral organs. (A) Inflorescence; (B) sepal; (C) petal; (D) pistil; (E) stamen. Scale bars: 2 mm (A), 200 µm (B–E).

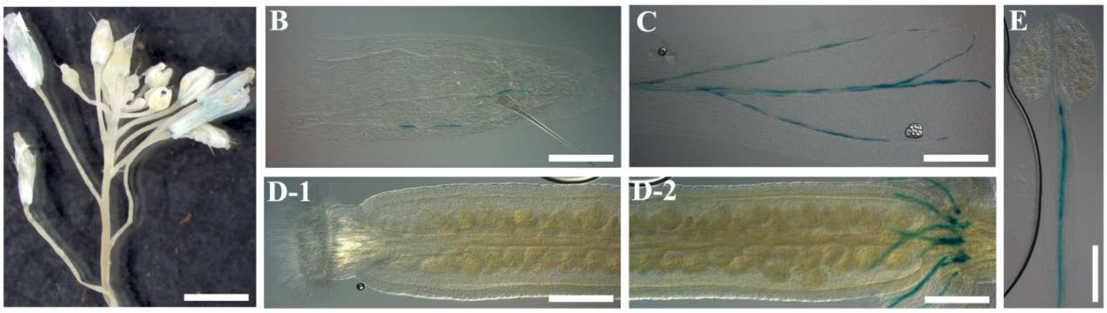

Supplementary Fig. S4. *AHP4pro*::*GUS* expression in Arabidopsis floral organs. (A) Inflorescence; (B) sepal; (C) petal; (D) pistil; (E) stamen. Scale bars: 2 mm (A), 200 μm (B–E).

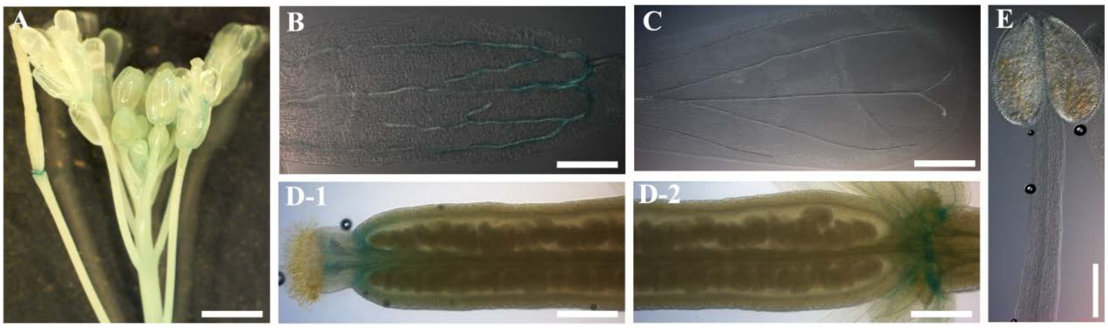

Supplementary Fig. S5. *AHP5pro*::*GUS* expression in Arabidopsis floral organs. (A) Inflorescence; (B) sepal; (C) petal; (D) pistil; (E) stamen. Scale bars: 2 mm (A), 200 μm (B–E).

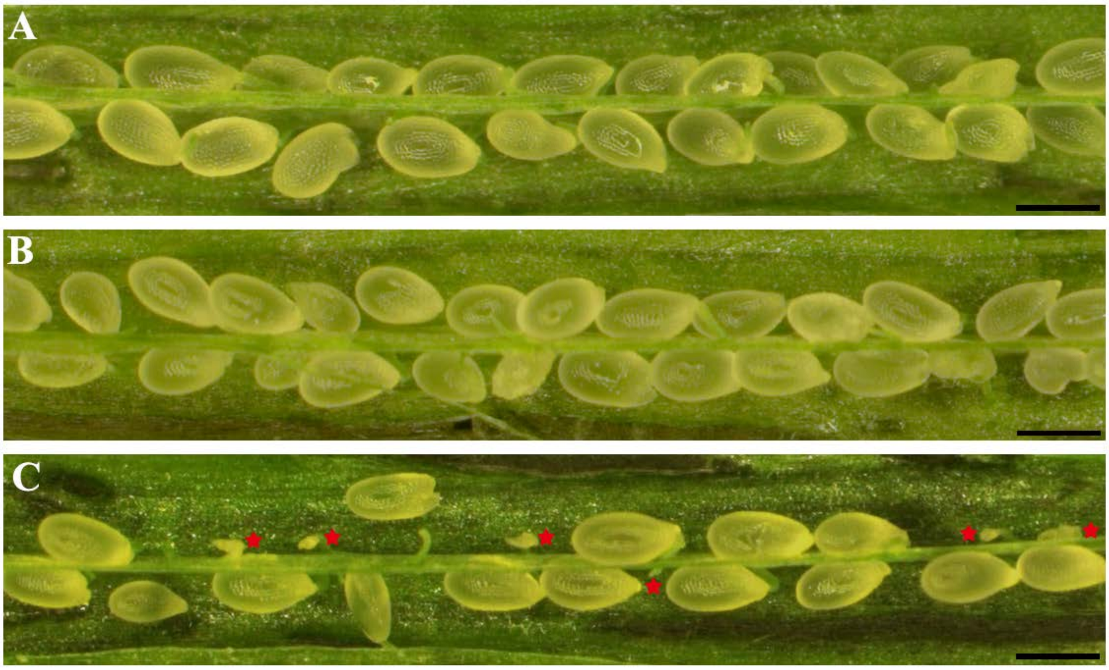

Supplementary Fig. S6. Microscopy of seed development in siliques. (A) Seed development in siliques of Wild type plant; (B) Seed development in siliques of *ahp2-*

2 ahp3 double mutant; (C) Seed development in siliques of *ahp2-2 ahp3 ahp5-2*/+ triple mutant, red asterisks indicate aborted seeds. Scale bars: 0.5 mm.

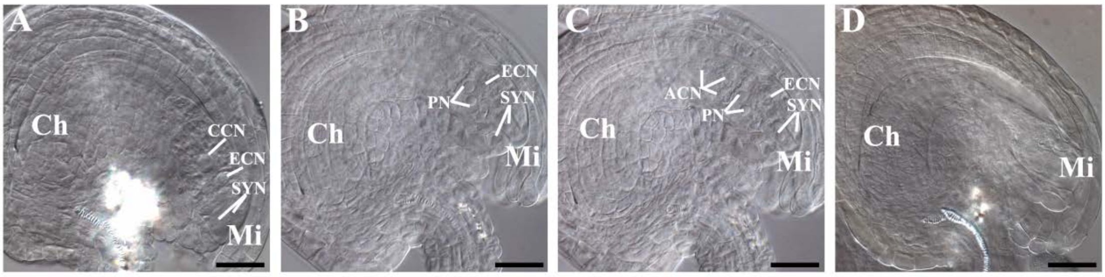

Supplementary Fig. S7. DIC microscopy of the cleared ovules in *ahp* multiple mutants. (A) Normal embryo sacs. (B–D) Abnormal embryo sacs. (B) Polar nuclei fail to fuse. (C) Polar nuclei fail to fuse; three antipodal cells persist without programmed cell death; antipodal cell nuclei move toward the micropylar end. (D) Degenerated embryo sacs with invisible female gametophyte nuclei. Ch, chalazal end; Mi, micropylar end; CCN, central cell nucleus; ECN, egg cell nucleus; SYN, synergid cell nuclei; ACN, antipodal cell nuclei; PN, polar nuclei. Scale bars: 15 µm.

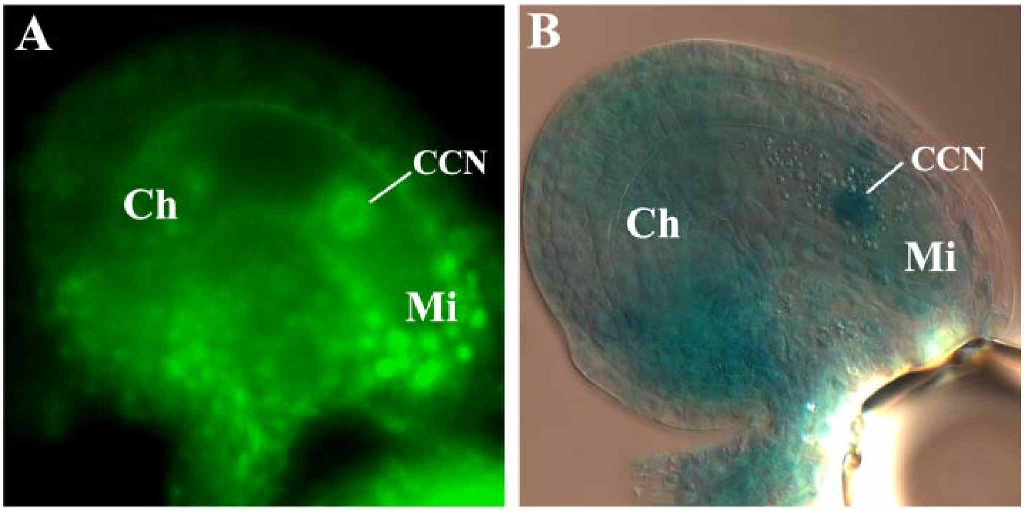

Supplementary Fig. S8. Transgenic expression in mature Arabidopsis embryo sacs. (A) *ETR1pro::NLS-3XeGFP* and (B) *ETR1pro*::*GUS*. Ch, chalazal end; Mi, micropylar end; CCN, central cell nucleus. Scale bars: 20 µm.
